# Evidence of Extended Thermo-Stability of Typhoid Polysaccharide Conjugate Vaccines

**DOI:** 10.3390/microorganisms9081707

**Published:** 2021-08-11

**Authors:** Fang Gao, Kay Lockyer, Alastair Logan, Sarah Davis, Barbara Bolgiano, Sjoerd Rijpkema, Gopal Singh, Sai D. Prasad, Samuel Pradeep Dondapati, Gurbaksh Singh Sounkhla

**Affiliations:** 1 National Institute for Biological Standards and Control (NIBSC), South Mimms EN6 3QG, UK; kay.lockyer@nibsc.org (K.L.); Alastair.Logan@nibsc.org (A.L.); sarah.davis8445@talktalk.net (S.D.); barbara.bolgiano@nibsc.org (B.B.); Sjoerd.Rijpkema@nibsc.org (S.R.); 2 Bharat Biotech International Limited (BBIL), Hyderabad 500078, India; gopal3176@bharatbiotech.com (G.S.); prasadsd@bharatbiotech.com (S.D.P.); samuel2816@bharatbiotech.com (S.P.D.); gurbaksh3875@bharatbiotech.com (G.S.S.)

**Keywords:** enteric, glycoconjugate, Hestrin, stability

## Abstract

Typhoid conjugate vaccines (TCV) are effective in preventing enteric fever caused by *Salmonella enterica* serovar Typhi in Southeast Asia and Africa. To facilitate vaccination with the Vi capsular polysaccharide–tetanus toxoid conjugate vaccine, Typbar TCV, and allow it to be transported and stored outside a cold chain just prior to administration, an extended controlled-temperature conditions (ECTC) study was performed to confirm the quality of the vaccine at 40 °C for 3 days at the end of its shelf-life (36 months at 2–8 °C). Studies performed in parallel by the vaccine manufacturer, Bharat Biotech International Limited, and an independent national control laboratory (NIBSC) monitored its stability-indicating parameters: *O*-acetylation of the Vi polysaccharide, integrity of the polysaccharide–protein conjugate, and its molecular size and pH. ECTC samples stored at 40 °C and 45 °C in comparison with control samples stored at 4 °C and 55 or 56 °C, were shown to have stable *O*-acetylation and pH; only very slight increases in the percentage of free saccharide and corresponding decreases in molecular size were observed. The deoxycholate method for precipitating conjugated polysaccharide was very sensitive to small incremental increases in percentage of free saccharide, in line with storage temperature and duration. This extended ECTC study demonstrated minimal structural changes to the Vi polysaccharide and conjugate vaccine and a stable formulation following extended exposure to elevated temperatures for the desired durations. This outcome supports the manufacturer’s ECTC claim for the vaccine to be allowed to be taken outside the cold chain before its administration.

## 1. Introduction

Supply of thermostable vaccines to control enteric fever in endemic regions of Asia and Africa would benefit country health programs, international organizations and vaccine producers. Enteric fever is caused by systemic infection with *Salmonella enterica* subspecies serovars Typhi (*S. Typhi*) and Paratyphi. It is a major contributor to the global disease burden with an estimated 9.24 million cases from *S. Typhi* in 2019 and 14.3 million from both *S. Paratyphi* and *S. Typhi* in 2017, contributing to approximately 1.53 million deaths per annum [1,2]. Whilst non-typeable *Salmonella* infections typically cause diarrheal illness, typhoid infections typically produce bacteremia accompanied by febrile illnesses, with prolonged high fever and headache being common symptoms [3]. These infections are relatively common in low- and middle-income countries (LMICs) with poor water supply and sanitation, but infections can be controlled through vaccination with typhoid conjugate vaccines. Vaccination can also break the escalating cycle of antibiotic resistance that is limiting the effectiveness of treatment options in areas with multidrug-resistant microorganisms [4]. 

Glycoconjugate vaccines, which use capsular polysaccharide (PS)-based components of encapsulated pathogens conjugated to epitopes of T cell-dependent protein antigens, are well-known for providing long-term immunity and the elimination of respiratory diseases in infants, children, and adults. The first demonstration of efficacy of a glycoconjugate vaccine against an enteric microorganism occurred with a typhoid Vi PS conjugated to recombinant exotoxin A of *Pseudomonas aeruginosa* [5]. The subsequent program of the World Health Organization (WHO) to strengthen the development of typhoid conjugate vaccines (TCVs) for vaccination programs led to manufacturing initiatives, human challenge trials, and field studies that have resulted in the rollout of vaccines in Africa and Southeast Asia [6,7,8,9]. Numerous vaccines have been licensed, including Typbar TCV (Bharat Biotech International Limited (BBIL), Hyderabad, India) in 2013, PedaTyph (BioMed, Ghaziabad, India) in 2008, and a TCV produced by Zydus Cadila (Ahmedabad, India) and licensed in 2017 [8], all of which consist of Vi PS, either from S. Typhi conjugated to tetanus toxoid (TT), conjugates with diphtheria toxoid [10], CRM_197_ (cross-reacting material 197, a non-toxic variant of diphtheria toxin [11]), and recombinant exotoxin A of *Pseudomonas aeruginosa* (rEPA) amongst others [12].

Typbar TCV received WHO prequalification in 2017, meaning it met the WHO expectations for quality, safety, and efficacy for procurement by the United Nations Children’s Emergency Fund (UNICEF) for use in global immunization programs [13]. To facilitate its supply to vaccination centers, the vaccine has also been subject to stability testing following the WHO protocol for extended controlled-temperature conditions (ECTC) [14] to ensure its quality and effectiveness if the cold chain cannot be guaranteed during the final stages of distribution. The current ECTC requirement is that the vaccine must exhibit a suitable stability profile following a single exposure to at least 40 °C for a minimum of 3 days at the end of its shelf-life. An application for the extension of a vaccine’s license (and label) can be made to allow for it to be taken out of the cold chain just prior to administration. This can facilitate greater flexibility for vaccination campaigns and reduce the demand for infrastructure requirements in the field, making this a real advantage for the supply and administration of TCVs in areas where typhoid is endemic. 

Key stability indicators for TCV that are required for ECTC study include the vaccine’s identity, molecular size and integrity of the conjugate (free Vi PS), *O*-acetylation content of Vi PS, and pH [12]. In this report, the manufacturer conducted a formal ECTC study and also collaborated with a control laboratory for independent evaluation of the vaccine’s thermostability. Following a preliminary ECTC collaborative study at 40 °C on single-dose vials of the TCV involving BBIL and the National Institute for Biological Standards and Control (NIBSC), the national control laboratory of the U.K., in 2015–2016, a further study was carried out on both single- and multi-dose formulations of the TCV vaccine to confirm its stability at 45 °C. This paper presents the results of that study and compares the methods used for its analysis. 

## 2. Materials and Methods

### 2.1. Materials 

Lots representing a single-dose and a five-dose Typbar TCV (Vi–TT conjugate) and a single-dose Typbar Vi PS vaccine (as control) were used by NIBSC in this study. All lots were taken from commercial batches that were near the end of their shelf-life (36 months at 2–8 °C). The manufacturer tested six lots of single-dose and 2 lots of multi-dose TCV. 

One single human dose (SHD) of the TCV and PS vaccine contains 25 ± 5 µg Vi PS from *S. Typhi*. The vaccine is presented in saline in the multi-dose vials, with 2-phenoxyethanol as a preservative [13].

### 2.2. Stability Conditions 

Vaccines were subjected to ECTC conditions at 35 months following their manufacture, one month before the end of their shelf-life. Samples were incubated at 40 ± 2 °C (BBIL) or 45 ± 2 °C (NIBSC) for 3 and 7 days. Vaccine vials were also stored at 2–8 °C (designated storage temperature) and 55 ± 2 °C (BBIL) or 56 ± 2 °C (NIBSC) as a high temperature control. Following exposure, all samples were stored at 2–8 °C until further analysis, which was completed before the end of shelf-life. 

### 2.3. Capture ELISA for Vi PS Identity 

A Vi PS capture ELISA was performed at NIBSC to determine the identity of Vi PS in typhoid vaccines, according to Hitri et al. [15] with some modifications. Following the coating of plates (Nunc Maxisorp) with horse anti-mouse IgG and a blocking step with 1% *w/v* BSA, TCV and Vi PS vaccine samples were diluted from 1:100 to 1:102,400 in assay buffer (PBS with 0.1% *v/v* Brij-35 (Thermo Scientific, Waltham, MA, USA 20150) and 1% *w/v* BSA) in 2-fold dilutions across the plate, with a final volume of 100 µL. Plates were incubated at room temperature for 1 h, washed, and 100 µL of rabbit anti-Vi serum (NIBSC 04/152) diluted 1:5000 in assay buffer was added to the wells and incubated at room temperature for 2 h. Plates were washed, and bound IgG was detected by incubation with 100 µL goat anti-rabbit IgG-HRP (Sigma, Kawasaki City, Japan) diluted 1:10,000 in assay buffer per well at room temperature for 1 h. Plates were developed, and ODs were read at 450 nm. CombiStats software was used to evaluate the binding curves. The sample identity is positive if the Vi PS content is calculated to be NLT 40 µg/mL based on the dose–response curve of the reference Vi PS preparation (NIBSC 16/126), and the curve should be comparable with NIBSC 16/126 with no significant deviations from parallelism or linearity.

### 2.4. Micro-Hestrin Assay of O-Acetylation 

The level of *O*-acetylation of Vi PS in the vaccine was quantified by the Hestrin method, a pharmacopeial assay. At NIBSC, a validated micro-Hestrin assay [15] based on Eur Ph 2.5.19 was performed using acetylcholine chloride (Sigma A-6625, purity NLT 99%) dissolved in 0.001 M sodium acetate with a range of 4.125 to 0.055 µmol/mL) as a standard. Standard and samples were analyzed in triplicate. The linear regression curve from the plot (µmol/mL versus optical density) gave the *O*-acetyl group content expressed as µmol *O*-acetyl/mL, which was converted into µmol/SHD. Assay precision (12.0% CV) was determined from two different Vi PS samples run in triplicate in two separate assays at NIBSC, and inter-laboratory precision between BBIL and NIBSC was 19.6% [16].

### 2.5. Molecular Sizing

At NIBSC, a Thermo (Dionex) ICS5000 System with Chromeleon software version 7.2 was used for molecular sizing analysis. An amount of 100 µL of TCV or PS vaccine was injected onto the Tosoh TSK 6000+5000 PWXL column series with a PWXL guard column (Tosoh Bioscience) and eluted with PBS ‘A’ (10.1 mM Na_2_HPO_4_, 1.84 mM KH_2_PO_4_, 171 mM NaCl, and 3 mM KCl, pH 7.3–7.5) at a flow rate of 0.25 mL/min for 130 min. The UV signals at 214 nm and 280 nm and the refractive index were monitored. The column oven was set to 30 °C. Consistency of elution of column calibrants was used as a system suitability test. The void elution time (determined with Salmon DNA, Sigma D1626) was at 47.9 min, and the total column elution time (tyrosine, Sigma T3754) was at 99.3 min using UV detection (280 nm). The distribution coefficient (K_D_) of the eluted peak and the percentage eluting by a specified K_D_ of 0.5 were determined using the 280 nm signal for TCV and 214 nm signal for the PS vaccine. The intermediate precision of the percentage eluting by a specified K_D_ was ±1.0. 

### 2.6. DOC Precipitation to Obtain Free Polysaccharide 

To separate the free Vi PS from the Vi–TT conjugate, a validated DOC-HCl precipitation method was used, based on the method of Lei et al. [17]. TCV was diluted to 10 µg Vi PS/mL in deH_2_O (MilliQ), and 100 µL of 1% *w/v* sodium deoxycholate (DOC, Sigma D6750) (pH 6.8) was added to 1 mL of sample to precipitate TT. The sample was incubated on ice for 30 min and 50 µL of 1 M HCl solution was added and then centrifuged at 6000× *g* at 22–23 °C for 15 min. The supernatant (containing free Vi PS) was removed, and samples were dried in a SpeedVac for 10 h before proceeding to the HPAEC-PAD assay. The percentage free Vi PS was calculated on the basis of measured total Vi saccharide content.

### 2.7. Vi Saccharide Content 

At NIBSC, Vi saccharide content was determined using the method of high-performance anion-exchange chromatography with pulsed amperometric detection (HPAEC-PAD). Vi PS from the procedure in 2.6 (free Vi) was reconstituted in 1 mL deH_2_O, alongside 1:5 diluted conjugate vaccine (total PS content), and hydrolyzed using NaOH (Fisher Scientific 10050470) at a final concentration of 2 M at 110 °C for 4 h [11]. Homologous Vi PS from *S. Typhi* IS (NIBSC 16/126) was used as reference, which is preferred over the use of heterologous Vi PS as a standard [16]. Vi PS from NIBSC 16/126 was diluted in water in a range from 27 to 0.5 µg/mL. A Thermo (Dionex) ICS5000 HPAEC-PAD system with the Amino Trap and CarboPac PA1 columns (Thermofisher Scientific, UK) was used for Vi PS quantitation. Eluting conditions were 0–2 min, 100 mM NaOH; 2–22 min, 100 mM NaOH and 40–150 mM NaNO_3_; and 22–31 min, 100 mM NaOH, with flow rate of 1 mL/min [11]. Vi was detected by pulsed amperometric detector with the following pulsed potential and durations: E1 = 0.1 V, t1 = 400 ms; E2 = −2 V, t2 = 20 ms; E3 = 0.6 V, t3 = 10 ms; E4 = −0.1 V, t4 = 60 ms. Chromeleon software (Version 7.2) was used to program the runs and analyze data. The data were converted into µg Vi PS/SHD by multiplying µg Vi PS/mL by 0.5 mL/SHD. The combined uncertainty of the method, factoring in method precision and reference standard uncertainty, was ±1.407 µg/dose.

At BBIL, Vi saccharide content was measured with a rocket immunoelectrophoresis method based on Szu et al. [18], using polysaccharide standards between 0.5 and 1.25 µg Vi and anti-Vi sera (rabbit polyclonal antibody, BD Difco) for detection. For further details, see Gao et al. [16].

### 2.8. pH Determination 

The pH of samples was determined at room temperature using a pH meter calibrated with pH 4, 7, and 10 buffers. The pH values were accurate to ±0.075 pH units at NIBSC and ±0.05 pH units at BBIL.

## 3. Results

### 3.1. Quality of the Polysaccharide 

*O*-acetylation of Vi polysaccharide is necessary for a protective immune response [19,20], and it is labile to extremes in pH and temperature. There was no indication of a decrease in *O*-acetylation levels in the single- or multi-dose vials during the stability study. Similar *O*-acetylation levels (*p* = 0.169) were obtained by both laboratories at initial release by the manufacturer and at end-of-shelf life by the control laboratory (Table 1, Table 2 and Table 3). There was no trend in *O*-acetyl level in ECTC samples, which were above the minimum limit recommended by WHO [21].

### 3.2. Integrity of the Conjugate

**Figure 1:**
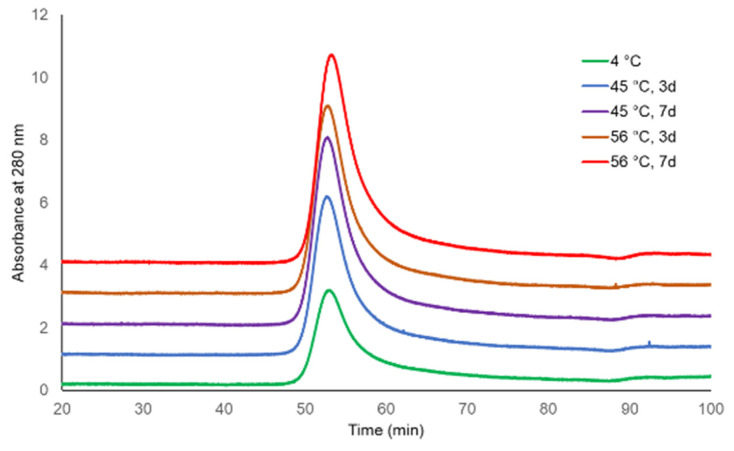
HPLC-SEC chromatograms of Typbar TCV single-dose stability samples (280 nm trace).

**Figure 2:**
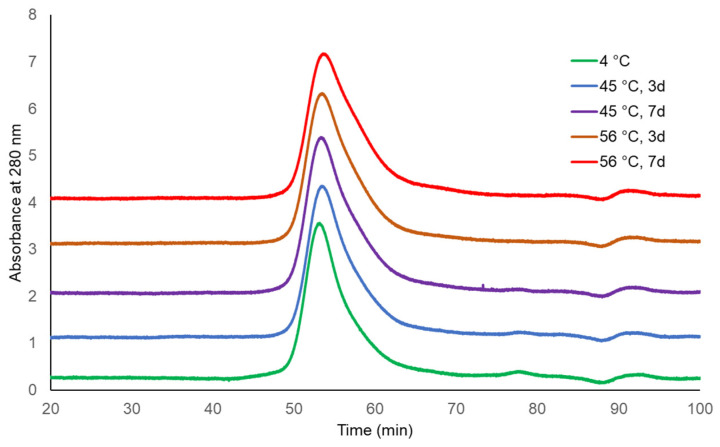
HPLC-SEC chromatograms of Typbar TCV multi-dose stability samples (280 nm trace).

**Figure 3:**
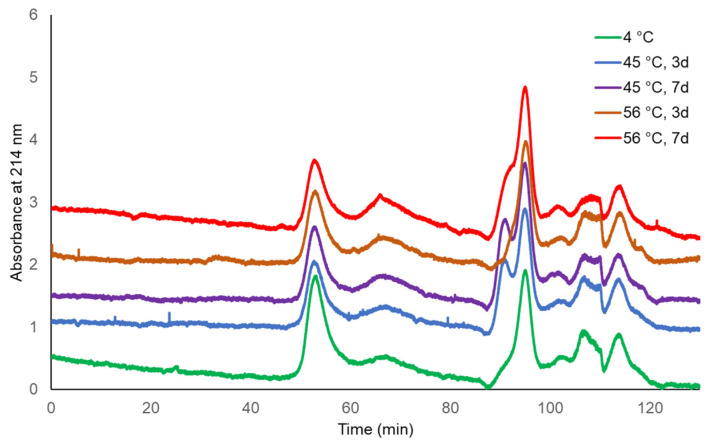
HPLC-SEC chromatograms of Typbar PS vaccine stability samples (214 nm trace).

The molecular sizing profiles of two lots of TCV were similar at both incubating temperatures and for both durations, with the exception of a slightly later eluting peak for TCV following storage at 56 °C for 7 days (Figure 1 and Figure 2, UV 280 nm). With a distribution coefficient of K_D_ = 0.5, 95.4% to 96.3% of the main peak eluted by the specified K_D_ for the single-dose formulation, as did 97.9% to 99.0% of the main peak of multi-dose TCV. The PS vaccine showed a lower percentage elution of Vi PS at K_D_ = 0.5 following storage at 56 °C (92.0%) compared with vaccine stored at 4 °C (96.3%) (Table 1 and Figure 3, UV 214 nm).

The percentage free saccharide for both single- and multi-dose TCV increased following storage at temperatures of 45 °C and 56 °C for 3 or 7 days, compared with vaccine stored at 4 °C, using the DOC-HPAEC-PAD method at NIBSC (Table 1). The amount of free saccharide increased at higher temperatures and with longer exposures, and the amount was compatible with 8% free saccharide detected at the end of shelf-life (2–8 °C). Changes were incremental, depending on temperature and incubation time. After 3 days at 56 °C, the amount of free saccharide rose to 11% and 13%, and after 7 days at 45 °C, free saccharide increased to 15% and 14% for the single-dose and multi-dose, respectively. Samples stored at 56 °C for 3 days were equivalent to those stored at 45 °C for 7 days, and the amount of free saccharide rose to 18% and 20% after 7 days storage at 56 °C for the single-dose and multi-dose, respectively. All samples exposed to elevated temperatures remained within 20% free saccharide (see Table 1). This confirmed the manufacturer’s data, which showed the same trend in all lots, either single- or multi-dose (Table 2 and Table 3). 

The pHs of all TCV samples were pH 6.9–7.1 at initial testing, and pH 6.6–7.2 following incubation at 40 °C or 45 °C for 3 days.

### 3.3. Vi Content in Stability Samples

The Vi PS content of single-dose Typbar TCV samples was within specification of 20–30 µg Vi PS/SHD across all ECTC conditions (40 °C or 45 °C). The exception was 31 µg/SHD for control sample stored at 56 °C for 7 days. The high Vi PS content of 36 µg/SHD determined in multi-dose sample stored at 4 °C was an outlier, but Vi content in all ECTC samples remained within specification, with a slight decrease among both temperatures with the duration of exposure.

The Vi PS content in Typbar polysaccharide vaccine increased at higher temperatures, but this was within the uncertainty of the method. A lower content was found after exposure at 56 °C for 7 days. This supplements the molecular sizing profile that indicated a deterioration in quality of the Vi PS stored at 56 °C for 7 days. All single-dose Typbar TCV and Typbar samples stored at 45 °C for 3 or 7 days were within the specification (Table 1).

Results from the manufacturer showed that the Vi PS contents in ECTC samples were within specification, without a trend of increase or decrease in those vaccines that were incubated in elevated temperature for different durations (Table 2 and Table 3).

## 4. Discussion

In a preliminary ECTC study, Typbar TCV was very stable following a 3-day exposure at 40 °C. Therefore, the stability of this vaccine was tested under harsher conditions (45 °C) with increased temperatures for exposure of up to 7 days, thus exceeding the WHO minimal requirement for an ECTC label claim, which is 3 days at 40 °C [14]. As sensitive stability indicators, pH and molecular sizing showed that Typbar TCV was very stable, except for samples exposed to 56 °C for 7 days, which both single- and multi-dose TCV had eluted slightly later compared with samples under other conditions, which indicated a small degree of degradation of the Vi PS polymer under such severe conditions.

As expected, both the temperature and the incubation time had an impact on the percentage free saccharide for TCV. The percentage free saccharide increased at higher temperature and after a prolonged exposure. Both NIBSC and the manufacturer used a similar DOC-precipitation method to measure free saccharide from conjugates, with results of 7.7% for the single-dose and multi-dose Typbar TCV at the end of shelf-life (NIBSC analysis), which was similar to the 5.7% and 5.1% free saccharide obtained by the manufacturer at the time of release. Although the manufacturer did not include exposure at 55 °C for 7 days as a condition, both datasets remained broadly comparable. The free saccharide content of the multi-dose was also equivalent to that of the single-dose at all conditions, which suggests the volume does not play a role in degradation. Different methods to separate free saccharide from the conjugate were tested at NIBSC, including ultrafiltration, SPE Vydac C4 cartridge (W.R Grace & Co. Columbia, MD, USA), and CaptoAdhere resin (GE Healthcare, Chicago, IL, USA) [22]. Although the C4 and CaptoAdhere resins showed some promise, they were not sensitive to slight changes in stability and were not suitable for analyzing the presence of free saccharide in Vi–TT conjugates in this ECTC study. On the contrary, good comparability between laboratories was observed with the DOC precipitation method, with expected increases in free saccharide with temperature and duration of incubation.

In a preliminary ECTC study at NIBSC, *C. freundii* Vi PS was used as a standard, and this resulted in an overestimation of the Vi PS content of the vaccine. Since then, *S. Typhi* Vi PS has become available as a reference [16], so this standard was subsequently used to quantify the Vi PS content in Typbar at NIBSC, and the manufacturer used an in-house Vi PS standard. Both laboratories measured similar values for the Vi PS content of Typbar TCV and Typbar stored at 4 °C. The manufacturer’s data showed Vi PS content consistent across all conditions for the five-dose vaccine and to be within specification.

The *O*-acetyl level is critical for Vi PS vaccines and it is directly related to the immunogenicity of the vaccine [19,20]. Hestrin assays were used by both laboratories to determine the levels of *O*-acetylation. The *O*-acetyl content in all ECTC samples remained within the limits recommended by WHO [21], and no trend was observed when assay precision was considered.

According to WHO guidelines, three lots must be evaluated for a valid ECTC study to demonstrate stability for 3 days at 40 °C. In parallel with evaluation of multiple lots by the manufacturer, the control laboratory evaluated one lot each of single- and multi-dose vaccine. The vaccine was found to be very stable, with little structural change of the Vi PS following exposure to temperatures at 40 to 45 °C for up to 7 days. Thus, we consider the quality of these vaccines to have been maintained following a single exposure at the end of shelf life. This observation should reduce vaccine wastage and greatly benefit the efficiency of the roll-out of TCV in endemic country programs.

## Figures and Tables

**Table 1:** Results of Typbar-TCV and Typbar samples under extended controlled-temperature conditions.

Sample	Storage		Identity	Molecular Size		Free Vi Content	Vi Content	*O*-Acetyl Content
Lot and age	Temp	Duration	By ELISA	Peak K_D_	% eluting by K_D_ = 0.5	% of total Vi PS	µg/SHD by HPAEC-PAD	µmole/SHD by Hestrin
Typbar TCV (Single-dose) tested at 35 months	+4 °C	3–7 day	+	0.10	95.4	7.7	28	0.089
	+45 °C	3 days	+	0.09	95.9	11.4	27	0.084
	+45 °C	7 days	+	0.10	95.8	14.6	27	0.090
	+56 °C	3 days	+	0.10	96.3	13.7	28	0.097
	+56 °C	7 days	+	0.11	95.4	17.5	31	0.093
Typbar TCV (Multi-dose) tested at 35 months	+4 °C	3–7 day	+	0.13	98.8	7.7	36	0.099
	+45 °C	3 days	+	0.13	99.7	12.6	30	0.101
	+45 °C	7 days	+	0.13	99.2	13.6	28	0.096
	+56 °C	3 days	+	0.13	99.3	14.8	27	0.090
	+56 °C	7 days	+	0.14	99.6	20.4	27	0.087
Typbar PS (Single-dose) tested at 35 months	+4 °C	3–7 day	+	0.10	96.0	n/a	25	0.090
	+45 °C	3 days	+	0.09	94.3	n/a	29	0.104
	+45 °C	7 days	+	0.09	96.3	n/a	30	0.084
	+56 °C	3 days	+	0.10	94.8	n/a	30	0.093
	+56 °C	7 days	+	0.09	92.0	n/a	18	0.075

**Table 2:** Results of Typbar-TCV single-dose samples under extended controlled-temperature conditions from the manufacturer.

Sample	Exposure		*O*-Acetyl Content	Vi Content	Free Vi Content
Lot no. and age	Temperature	Duration	µmoles/SHD by Hestrin	µg/SHD by Rocket	% of total Vi PS
76DL16001, Day 0	+4 °C	0 day	0.093	27	4.4
76DL16001, 35 Months	+40 °C	3 days	0.089	27	11.3
76DL16001, 35 Months	+40 °C	7 days	0.084	26	13.5
76DL16001, 35 Months	+55 °C	3 days	0.086	28	16.7
76DL16002, Day 0	+4 °C	0 day	0.098	27	5.0
76DL16002, 35 Months	+40 °C	3 days	0.086	27	10.7
76DL16002, 35 Months	+40 °C	7 days	0.082	28	11.8
76DL16002, 35 Months	+55 °C	3 days	0.085	28	14.9
76DL16003, Day 0	+4 °C	0 day	0.095	27	4.1
76DL16003, 35 Months	+40 °C	3 days	0.087	26	12.8
76DL16003, 35 Months	+40 °C	7 days	0.083	27	13.7
76DL16003, 35 Months	+55 °C	3 days	0.087	27	17.9
76DL16033, Day 0	+4 °C	0 day	0.085	28	5.6
76DL16033, 35 Months	+40 °C	3 days	0.089	28	10.5
76DL16033, 35 Months	+40 °C	7 days	0.089	29	11.2
76DL16033, 35 Months	+55 °C	3 days	0.086	28	11.8
76DL16034, Day 0	+4 °C	0 day	0.073	28	4.8
76DL16034, 35 Months	+40 °C	3 days	0.080	28	9.3
76DL16034, 35 Months	+40 °C	7 days	0.082	28	9.8
76DL16034, 35 Months	+55 °C	3 days	0.078	28	10.4
76DL16035, Day 0	+4 °C	0 day	0.081	28	5.1
76DL16035, 35 Months	+40 °C	3 days	0.081	28	11.5
76DL16035, 35 Months	+40 °C	7 days	0.080	28	11.9
76DL16035, 35 Months	+55 °C	3 days	0.076	28	12.6

**Table 3:** Results of Typbar-TCV multi-dose samples under extended controlled-temperature conditions from the manufacturer.

Sample	Exposure		*O*-Acetyl Content	Vi Content	Free Vi Content
Lot no. and age	Temperature	Duration	µmoles/SHD by Hestrin	µg/SHD by Rocket	% of total Vi PS
76CJ16003, Day 0	+4 °C	0 day	0.098	29	5.9
76CJ16003, 35 Months	+40 °C	3 days	0.097	27	10.9
76CJ16003, 35 Months	+40 °C	7 days	0.096	28	11.7
76CJ16003, 35 Months	+55 °C	3 days	0.095	27	11.5
76CJ16004, Day 0	+4 °C	0 day	0.104	28	4.2
76CJ16004, 35 Months	+40 °C	3 days	0.099	28	12.3
76CJ16004, 35 Months	+40 °C	7 days	0.098	28	12.9
76CJ16004, 35 Months	+55 °C	3 days	0.098	28	12.6

## Data Availability

The data presented in this study are available on request from the corresponding author.
